# Supplementary material for: Anatomical ablation for small hepatocellular carcinomas using multiple applicators: a preliminary study
Source: Cancer Imaging. 2023 Aug 21;23:78. doi: 10.1186/s40644-023-00597-0 (PMC10440891; doi:10.1186/s40644-023-00597-0)
Supplement: Supplementary file 1 — Supplementary Material 1 [file 40644_2023_597_MOESM1_ESM.docx]

**Anatomical Ablation for Small Hepatocellular Carcinomas Using Multiple Applicators: A Preliminary Study**

R2-1

Jae Hyun Kim, M.D.^a^, Hee Soo Kim, M.D.^a^, Jeong Hee Yoon, M.D.^a^, Ijin Joo, M.D.^a^, Jung-Hwan Yoon, M.D.^b^, Yoon Jun Kim, M.D.^b^, Su Jong Yu, M.D.^b^, Jeong Min Lee, M.D.^a,c^

^a^ Department of Radiology, Seoul National University Hospital, Republic of Korea

^b^ Department of Internal Medicine, Seoul National University Hospital, Republic of Korea

^c^ Institute of Radiation Medicine, Seoul National University Medical Research Center, Seoul, Republic of Korea

**Corresponding Author:** Jeong Min Lee, M.D.

Department of Radiology, Seoul National University Hospital, 101, Daehak-ro, Jongno-gu, Seoul, Republic of Korea, 03080.

Tel: 82-2-2072-3154; Fax: 82-2-743-6385; E-mail: [jmsh@snu.ac.kr](mailto:jmsh@snu.ac.kr)

**ABSTRACT**

**Background:** Anatomical ablation, defined as thermal ablation of tumor-bearing small portal territories, may provide excellent local tumor control in peripherally-located small hepatocellular carcinomas (HCC), which has been a major concern with percutaneous ablation alone.

**Purpose:** To evaluate the technical feasibility and therapeutic outcomes of anatomical ablation using multiple radiofrequency (RF) applicators for the ablation of tumor-bearing small portal territories of peripherally-located small (≤4 cm) HCCs.

**Materials and Methods**: Patients with peripherally-located single HCCs (≤4 cm) to be treated with anatomical ablation using multiple RF applicators between January 2020 and March 2022 were enrolled in this prospective study. Anatomical ablation was performed for the index tumor under real**-time US-CT/MR fusion imaging guidance, with one or two clustered electrode needles placed across the tumor-bearing portal vein branches. Technical success and complications of anatomical ablations were assessed.** Cumulative incidence of local tumor progression (LTP) and recurrence-free survival were estimated using the Kaplan–Meier method.

**Results:** Fifty-five HCCs (mean size, 1.77±0.59 cm) in 55 participants (mean age, 66.4±7.7 years; 39 men, 16 women) were treated with anatomical ablation; 98.2% (54/55) technical success was achieved. **No major complications were noted.**Among the 55 participants, LTP occurred in only one patient who had experienced technical failure of anatomical ablation. Estimated 1- and 2-year cumulative incidences of LTP were 0% and 3.7%, respectively. Five patients developed intrahepatic remote recurrence during the median follow-up period of 19.2 months (range, 3.7–28.8 months); therefore, estimated 1- and 2-year recurrence-free survival was 91.7% and 85.0%, respectively.

**Conclusion:** Anatomical ablation using multiple RF applicators provided the excellent results of local tumor control in patients with peripherally-located small (≤4 cm) HCCs.

**Trial registration:** *clinicaltrial.gov* identifier: NCT05397860

**Keywords:** Tumor ablation; Multiple applicators; Image fusion; Anatomical ablation; Hepatocellular carcinoma

**INTRODUCTION**

Hepatocellular carcinoma (HCC) is one of the leading causes of cancer-related death worldwide ([1](#_ENREF_1)). Until now, image-guided radiofrequency ablation (RFA) or microwave ablation (MWA) has been widely accepted to be an effective curative treatment option for early stage HCCs smaller than 3 cm in diameter and is suggested in guidelines proposed by several of the major international societies ([2-4](#_ENREF_2)). Nonetheless, the results of percutaneous ablation remains unsatisfactory compared to those of surgical resection particularly in terms of local tumor progression (LTP) ([4-8](#_ENREF_4)), which is one of the most important independent factors for recurrence free survival and overall survival after ablation ([7](#_ENREF_7)). With several studies reporting that the development of LTP after ablation is affected by the size of the ablative margin, a 0.5–1.0 cm ablative margin has generally been pursued for liver tumor ablation ([9-12](#_ENREF_9)). Recent studies have also demonstrated that the creation of a large ablative margin greater than 1.0 cm using MWA may help further reduce LTP ([13](#_ENREF_13), [14](#_ENREF_14)). Regardless of the ablative margin, however, it is generally accepted that percutaneous ablation using the conventional tumor puncture technique has inherent limitations in controlling microscopic metastases originating from the primary tumor through microscopic vascular invasion compared with surgical resection ([15](#_ENREF_15), [16](#_ENREF_16)). Indeed, in a previous study of 149 resected specimens in patients with small HCCs (≤ 3cm), 9.5% of HCCs were shown to have satellite lesions located 0.5–1.0 cm from the main tumor, and 3% within 1.0–2.0 cm ([17](#_ENREF_17)). In addition, microvascular invasion of the portal vein was found in up to 33.3% of cases when the HCC was between 2 cm and 3 cm in size ([17](#_ENREF_17), [18](#_ENREF_18)).

Recently, based on previous studies on hepatic angiography which have demonstrated that the drainage veins of progressive HCCs are the portal venules rather than hepatic veins, and that HCC tends to metastasize through the portal vein, often manifesting as satellite nodules within the venous drainage area of the primary tumor ([19](#_ENREF_19), [20](#_ENREF_20)), it has been hypothesized that early blockage of the draining portal vein during ablation or resection of the hepatic parenchyma of the tumor-bearing segment fed by the portal branches could be considered a logical method of eliminating potential intrahepatic metastases ([21](#_ENREF_21)). Indeed, surgical studies have already demonstrated that systematic removal of tumor-bearing portal territories could reduce the risk of tumor recurrence and improve patient survival, correlating with decreased local recurrence ([22-26](#_ENREF_22)). Thus, with new technological developments in ablation equipment and guiding modalities, including a third generation microwave system, high powered multi-channel RF generators, multi-bipolar or separable clustered electrodes, and real-time US-multimodality fusion imaging ([26](#_ENREF_26), [27](#_ENREF_27)) which can be applied for anatomical ablation similar to subsegmental anatomical resection ([25](#_ENREF_25), [26](#_ENREF_26)), we hypothesized that with multiple applicators and a high powered (~400W) multi-channel generator, one applicator can be used to ablate tertiary or 4^th^ portal vein branches near the index tumor, and the other applicators can be used to ablate the index tumor and surrounding parenchyma with more effective RF or MW energy delivery. In addition, a recently published retrospective study demonstrated that anatomical thermal ablation could be a favorable ablation strategy to treat HCC compared with routine thermal ablation ([28](#_ENREF_28)).

R2-2

Therefore, this study aimed to prospectively investigate the technical feasibility and therapeutic outcomes of anatomical ablation using multiple RF applicators to ablate tumor-bearing small portal territories of peripherally-located small (≤4 cm) HCCs so as to reduce local tumor progression after ablation.

**MATERIALS AND METHODS**

**Compliance with Ethical Standards**

This single-center, prospective study was approved by the Institutional Review Board of Seoul National University Hospital (IRB No. H-1909-086-1064) and written informed consents were obtained from all enrolled participants (*clinicaltrial.gov* identifier: NCT05397860).

**Study Population**

This single-center, single-arm, prospective interventional study evaluated participants with small nodular HCCs (≤4 cm) between January 2020 to March 2022 for potential enrollment in the study. The inclusion criteria were as follows: a) contrast-enhanced CT or MRI within 60 days prior to RFA; b) single HCCs, 1.0–4.0 cm; c) Child–Pugh class A or B liver function; and d) age, 20–85 years. The exclusion criteria for this study were as follows: a) two or more tumors; b) largest tumor size >4 cm; c) tumors with macrovascular invasion and/or distant metastasis; d) a platelet count of less than 50,000 mm^3^, or international normalized ratio greater than 1.5 (prothrombin time >1.5 times normal); or e) Child–Pugh class C.

**Ablation Procedures**

For the diagnosis of HCCs, noninvasive imaging criteria according to the Korean Liver Cancer Association-National Cancer Center Korea guidelines ([29](#_ENREF_29)) were used. Regarding anatomical ablation, 4^th^ or 5^th^ portal vein branches near the tumor to be included in the ablation zone were identified using preprocedural multiphasic liver CT or gadoxetic acid-enhanced liver MRI. All ablation procedures were performed percutaneously under conscious sedation by a single attending radiologist with 26 years of experience and a resident or a clinical fellow. Real-time multimodal US fusion imaging (RS 85, Samsung Medison) was used as the guidance modality. Among the 55 patients, SonoVue (Bracco, Milan, Italy) and/or Sonazoid (GE Healthcare, Milwaukee, Wis, USA) were used in 50 patients to help with accurate tumor localization and ablation procedure guidance. A separable clustered electrode with three electrodes (Octopus RF electrode; STARmed) and a multichannel RF generator (VIVA RF System; STARmed) with a maximum power of 400 W (2 amps of 200W) were used. All HCCs located in the peripheral portion of the liver (within 5 cm of the capsule) were treated using anatomical ablation. In addition, depending on the presence of sufficient peritumoral liver tissue for placement of the electrodes or the availability of a safe access route for multiple electrode insertions required for no-touch ablation, the operator chose between no-touch ablation or conventional tumor puncture ablation. The technical success of anatomical ablation, defined as the combined ablation of the index tumor and a sufficient safety margin (>5 mm) was then recorded.

***Anatomical ablation.*** —The number and active needle tip length of the electrode were chosen based on the tumor size, as described in our previous study ([30](#_ENREF_30)). When the tumor size was <1.5 cm, two or three electrodes with 2-cm active tips were selected, whereas for 1.5–4.0 cm-sized tumors, three electrodes with 2.5- or 3-cm active tips were used. For anatomical ablation, one or two electrodes were placed across the 4^th^ or 5^th^ subsegmental branches of the portal vein ≤2 mm near the index tumor (Fig. 1). The target portal vein branch was determined by referring to the CT or MR images from multimodal US fusion imaging and color/power Doppler or microvascular flow images or contrast-enhanced US ([31](#_ENREF_31)). The other electrodes were placed either within the tumor (conventional tumor puncture technique) or peritumoral zone (no-touch technique) depending on the technique used. When there was sufficient peritumoral liver parenchyma and a safe access route, the no-touch technique was preferentially applied ([30](#_ENREF_30), [32](#_ENREF_32)). For no-touch ablation, all electrodes were inserted outside the target tumor under real-time fusion US-MR/CT guidance, as described previously ([30](#_ENREF_30), [33](#_ENREF_33)). When there was no safe access route for the insertion of multiple electrodes, we used the conventional tumor puncture method, and the electrodes were inserted in the central portion of the tumor ([34](#_ENREF_34)). Interelectrode distance was adjusted according to the target tumor size and was maintained at 2–3 cm. After insertion of the electrodes, RF energy was delivered to two of the three electrodes simultaneously using an automatic gradual incremental technique from 60 to 200 W for 8–12 min. Ablation time was adjusted according to the tumor size and number of inserted electrodes. Thereafter, creation of an echogenic complex was carefully monitored under real-time fusion US-MR/CT guidance. When the operator determined that the echogenic complex showed an insufficient safety margin (≤5 mm) around an electronic “virtual” target of real-time fusion US-MR/CT after the initial ablation, repositioning of the electrode was performed to obtain a sufficient safety margin ([35](#_ENREF_35)).

Immediately after ablation, contrast-enhanced liver CT was performed in all participants to assess the technical success of anatomical ablation and to detect any potential complications related to the ablation procedures, such as bleeding. **Technical success of anatomical ablation was defined as combined ablation of an index tumor with a sufficient safety margin (>5 mm) around the tumor as well as obliteration of the adjacent portal vein 4^th^ branches.** If a residual tumor was to be identified on immediate CT scans, additional ablation was to be performed to achieve technical success. However, once complete ablation of the target tumor was attained, additional ablations were not to be performed, regardless of whether obliteration of the adjacent portal vein 4^th^ branches was securely obtained. The development of post-ablation complications as well as the duration of the hospital stay were also evaluated through a review of the medical records and imaging studies. Post-ablation complications were graded according to Clavien-Dindo classification ([36](#_ENREF_36)). Complications of grade IIIa or higher were considered as major complications and the rest were considered as minor complications.

**Technique efficacy, local control, and progression assessment**

For each patient, the first follow-up contrast-enhanced CT or MRI with alpha-fetoprotein serum test was undertaken after 1 month. We defined technique efficacy as complete ablation of the index tumor. Patients with complete ablation of the index tumor at the 1-month follow-up imaging were followed up with contrast-enhanced CT or MRI and serum alpha-fetoprotein every 3 months. The primary efficacy rate refers to the percentage of target tumors successfully ablated following the initial ablation, whereas the secondary efficacy rate refers to the percentage of index tumors finally eradicated with repeated ablation. Local control is equivalent to secondary technique efficacy, with the exception of repeated treatments using alternative methods such as transarterial chemoembolization ([37](#_ENREF_37)). Patients with successful treatments were followed with contrast-enhanced CT or MRI and serum alpha-fetoprotein every 3 months thereafter. HCC recurrence after ablation was categorized into three groups: a) LTP, defined as the appearance of tumor foci at the margin of the ablation zone after the attainment of treatment success; b) intrahepatic remote recurrence, defined as the presence of HCC in the liver at a site discontinuous to the ablation zone; and c) extrahepatic metastasis ([11](#_ENREF_11)).

**Statistical Analysis**

The cumulative incidence of LTP, intrahepatic remote recurrence, and recurrence-free survival were estimated using the Kaplan–Meier method. All statistical analyses were performed using MedCalc software (MedCalc version 20.0.23; MedCalc Software, Mariakerke, Belgium).

**RESULTS**

R1-4

A total of 55 patients were enrolled in this study (Fig. 2). No-touch ablation which refers to ablation without violating the tumor was performed in 29 participants (52.7%) and tumor puncture ablation was performed in 26 participants (47.3%). The mean tumor diameter was 1.77±0.59 cm. The baseline characteristics of participants are summarized in Table 1.

**Technical success of anatomical ablation and post-ablation complications**

Anatomical ablation was performed with a success rate of 98.2 % (54/55) (Figs. 2 and 3). In the case of technical failure of anatomical ablation, ablation of a subsegmental portal vein branch located near the medial border of the index tumor had failed (Fig. 4). Mean ablation time per procedure was 9.29±3.63 minutes; mean ablation diameter was 5.34±0.95 cm. All patients (n=55) showed complete ablation of the index tumor on immediate CT scans. No patient underwent repeated ablation.

Among the 55 patients treated by anatomical ablation, three patients experienced minor complications (5.5%, 3/55): grade I (fever, n=3). No major complications or procedure-related deaths occurred. Moreover, no cases were observed in which thrombosis extended from the targeted portal vein 4th branch to the proximal portal vein or led to liver failure due to large portal vein thrombosis. Mean hospital stay was 1.3±1.2 days (range, 1–8 days).

R2-3

**Technique efficacy and recurrence outcomes: LTP and recurrence-free survival**

All participants attained complete ablation of the index tumor as evaluated at the 1-month follow-up CT or MRI (primary efficacy, 100% [55/55]).

During a median follow-up of 19.2 months (range, 3.7–28.8 months), LTP occurred in one of the 55 participants, and the recurrent tumor was treated using transarterial chemoembolization. In the participant with LTP, both anatomical ablation and no-touch ablation had failed, and an enhancing tumor occurred at the subsegmental portal vein branch where the ablation had failed (Fig. 4). In contrast, in all cases in which anatomical ablation was successfully performed, there were no incidences of LTP or intrasegmental recurrence. According to the intention-to-treat analysis, the estimated cumulative incidence of LTP in anatomical ablation with or without technical success at 1 and 2 years was 0% and 3.7%, respectively (Fig. 5).

The estimated cumulative incidence of intrahepatic remote recurrence after anatomical ablation at 2 years was 11.3%. Additionally, there were no cases of extrahepatic metastasis or intrasegmental recurrence in the tumor-bearing segment after anatomical ablation during the follow-up period. The estimated recurrence-free survival rates at 1 and 2 years following anatomical ablation were 91.7% and 85.0%, respectively (Fig. 5). Regarding participants in whom technical success of anatomical ablation was achieved (n=54), the estimated recurrence-free survival rates at 1 and 2 years were 91.5% and 88.4%, respectively.

**DISCUSSION**

In this prospective study, we assessed the feasibility and treatment outcomes of real-time fusion imaging-guided anatomical ablation of the portal territories of peripherally located small HCCs smaller than 4 cm using multiple applicators. **Percutaneous ablation was conducted using a multichannel RF generator and separable clustered electrodes in dual-switching monopolar mode up to 400W in maximum** under r**eal-time US-CT/MR fusion imaging guidance. We had hypothesized that, if technically successful, local tumor control may be improved** as early blockage of the draining portal vein branches near the index tumor and ablation of the tumor-bearing subsegments would help to limit intrahepatic metastases. Indeed, out of 55 participants, all but one patient was able to achieve technical success in anatomical ablation, and none of the participants demonstrated LTP or intrasegmental recurrence after a mean follow-up of 21 months. In fact, the only case of LTP in our study had occurred in the sole case of technical failure, at the remnant subsegmental portal vein branch which was unable to be ablated. Thus, the estimated 1- and 2-year cumulative incidence of LTP was shown to be 0% and 3.7%, respectively. According to a recently published retrospective study ([28](#_ENREF_28)), the anatomical ablation group exhibited a 3-year LTP rate of 0%. However, the study did not provide specific information on the success rate of anatomical ablation, including complete ablation of tumor-bearing portal vein branches. It is conceivable that in the anatomical ablation group, portal vein branch ablation was successful in all patients, resulting in a lack of LTP cases. In parallel, our study also demonstrated a lack of LTP cases when anatomical ablation was successful, implying comparable outcomes. Previous studies had demonstrated that local control of the tumor after ablation therapy or transarterial chemoembolization is the key to prolonging the prognosis of patients with HCC ([38](#_ENREF_38), [39](#_ENREF_39)). Therefore, we believe that with the excellent results of local tumor control or intrasegmental recurrence achieved after anatomical ablation, this technique may hold great promise in the management of patients with very early or early stage HCCs.

R2-6

The excellent local tumor control rates achieved using the anatomical ablation system in our study could be attributed to several oncologic and thermal advantages. First, as we placed one or two electrodes near the draining portal vein of the index tumor, early venous drainage, hence the dispersal of tumor cells into the drainage bloodstream may have been blocked. This concept is similar to the technical aspects of ultraselective transarterial chemoembolization with lipiodol, which induces lipiodol reflux into the draining portal venule ([40](#_ENREF_40)). Second, as the draining portal vein is the supplying vein of the tumor-bearing subsegment, early blockage of the portal blood supply would have reduced the heat-sink effect thereby improving the thermal efficiency of parenchymal ablation around the tumor. Third, creation of a larger safety margin (> 5mm) around the target tumor using a high-powered multichannel RF system (~400W maximum RF current with two 200W generators) with blockage of venous outflow must be advantageous to ablate microscopic peritumoral tumor deposits and microvascular invasion. Indeed, a previous pathologic study demonstrated that single nodular type with extranodular growth and confluent multinodular type showed a higher frequency of microvascular invasion than 20% and a longer distance between the main HCC and intrahepatic metastases than 5 mm ([17](#_ENREF_17)). As there was no intrasegmental recurrence in the 54 patients in whom anatomical RFA was successfully applied, anatomical ablation can be a more effective treatment option than conventional ablation for peripherally located small HCCs, especially when tumors show irregular margins or confluent multinodular shapes. Similarly, we expect that anatomical ablation could be more effectively achieved with multi-channel MW system and multiple applicators under guidance of contrast-enhanced CT because MWA is associated with higher intratumoral temperatures, resulting in less heat-sink effect compared to RFA ([41](#_ENREF_41)). Further study is needed to evaluate the feasibility of anatomical ablation using multiple microwave applicators.

Of technical note, we used multimodal US fusion imaging technology with either color Doppler or microvascular flow imaging techniques or contrast-enhanced US to identify the portal vein anatomy in the tumor-bearing segment ([26](#_ENREF_26), [31](#_ENREF_31), [42](#_ENREF_42)). To achieve anatomical ablation, precise placement of the electrode near the draining portal vein or passing through the vein must be performed. Kang et al. ([31](#_ENREF_31)) had previously demonstrated that microvascular flow imaging which can separate slow or small-vessel flow signals from clutter artifacts that arise from voluntary and involuntary motion by using a vendor-specific adaptive filter could detect the slow flow of small tumor vessels of HCCs better than the other Doppler techniques. In our study, the combined use of fusion imaging and microvascular flow imaging technique proved valuable in demonstrating the portal vein anatomy in the tumor bearing segment, and aided in electrode placement for the draining portal venule. Fusion imaging was also shown to be valuable for ablation procedure monitoring, making the anatomical ablation technique easier to perform ([26](#_ENREF_26), [42-44](#_ENREF_42)).

In our study, 98.2% (54/55) of participants achieved technical success following anatomical ablation, and the estimated 1- and 2-year cumulative incidences of LTP were 0% and 3.7%, respectively. Theoretically, acquiring a sufficient margin (5–10 mm) around the index tumor after ablation and early blockage of portal venous drainage could be critical for decreasing LTP or intrasegmental recurrence ([10](#_ENREF_10), [45](#_ENREF_45), [46](#_ENREF_46)). Anatomical ablation using multiple applicators could be used with either conventional tumor puncture (47.3%) or “no-touch” approach (52.7%). Recent studies on “no-touch” RFA, have shown satisfactory local tumor control rates as low as 4% at 3 years ([47-50](#_ENREF_47)), which could be attributed to easier creation of a sufficient ablation margin and no elevation of intratumoral pressure during ablation ([51](#_ENREF_51), [52](#_ENREF_52)). Although combination of no-touch ablation and anatomical ablation, may maximize the oncological benefits of each technique, in clinical practice, no-touch ablation may not be possible in patients with peripherally located small HCC who lack a sufficient peritumoral parenchyma (<5 mm width around more than half portion of tumor) or a safe access route ([30](#_ENREF_30)). In our study, no intrasegmental metastases was developed after anatomical ablation regardless of tumor puncture or no-touch approach used. Considering that the LTP rate was 8.5% despite securing sufficient ablation margin by using the dual-switching monopolar mode in a previous study ([34](#_ENREF_34)), we believe that early blockage of blood flow in the portal venule around the index tumor may contribute for reducing intrasegmental metastases after conventional tumor puncture ablation.

Our study had several limitations. First, the follow-up duration after ablation (median, 19.2 months) was relatively short. Second, the effectiveness of ablation may depend on operator experience and skill. Ablations were performed by an experienced radiologist at a high-volume center in our study, and therefore, further multicenter study with several operators having variable experiences would be warranted to confirm reproducibility of our study results. Third, we used only one high powered multichannel RF system and separable cluster electrodes in switching monopolar mode. Further studies are required to determine whether the same results can be achieved with other RF devices or other thermal sources, such as microwave ablators. However, considering better thermal efficiency of MWA than RFA, we expect that MWA using multi-channel MW systems with multiple antennas could produce even better study results in terms of local tumor control. Lastly, some of the participants had undergone prior HCC treatment for other HCC, although patients had no recurrence for two years from the initial treatments. Further study in patients with de novo early stage HCC would be necessary to evaluate overall survival after anatomic ablation.

**CONCLUSION**

In conclusion, anatomical ablation using multiple RF applicators may help improve local tumor control and is a promising treatment method for peripherally-located small (≤4 cm) HCCs.

**ABBREVIATIONS**

HCC = hepatocellular carcinoma

RFA = radiofrequency ablation

MWA = microwave ablation

US = ultrasonography

CT = computed tomography

MR = magnetic resonance

LTP = local tumor progression

**DECLARATIONS**

**Ethical Approval and Consent to participate**

This study was conducted ethically in accordance with the World Medical Association Declaration of Helsinki and approved by the Institutional Review Board of Seoul National University Hopsital (IRB No. H-1909-086-1064). All enrolled participants provided written informed consent (*clinicaltrial.gov* identifier: NCT05397860).

**Consent for publication**

Not applicable.

**Availability of data and materials**

The datasets used and/or analysed during the current study are available from the corresponding author on reasonable request.

**Competing interests**

There are no relevant conflicts of interest related to the submitted work. J.M.L has received grants from Bayer Healthcare, Canon Healthcare, Philips Heathcare, GE Healthcare, CMS, Guerbet, Samsung Medison, and Bracco. J.M.L has received personal fees from Bayer Healthcare, Siemens Healthineer, Samsung Medison, Guerbet, and Philips Healthcare. J.H.Y has received honorarium from Bayer Healthcare and personal fee from Philips Healthcare. For the remaining authors none were declared.

**Funding**

**This study received technical support and was supported by a research grant (grant number 06-2016-0590) from STARmed Co., and the Korea Medical Device Development Fund grant funded by the Korea government (the Ministry of Science and ICT, the Ministry of Trade, Industry and Energy, the Ministry of Health & Welfare, the Ministry of Food and Drug Safety) (Project Number: RS-2020-KD000303, 1711138983 )**

**Authors’ contributions**

 Conceptualization: J.M.L

 Data curation: J.H.K

 Formal analysis: J.H.K

 Funding acquisition: N/A

 Investigation: J.M.L

 Methodology: J.M.L, J.H.Y, I.J, J.H.K, H.S.K

 Project administration: J.M.L

 Resources: J.M.L

 Software: N/A

 Supervision: J.M.L, J.H.Y, I.J, J.Y, Y.J.K, S.J.Y

 Validation: N/A

 Visualization: J.H.K.

 Writing-original draft: J.M.L, J.H.K

 Writing-review & editing: J.M.L, J.H.Y, I.J, J.H.K, H.S.K, J.Y, Y.J.K, S.J.Y

**Acknowledgements**

Not applicable

**REFERENCES**

1. Villanueva A. Hepatocellular Carcinoma. N Engl J Med. 2019;380(15):1450-62.

2. Omata M, Cheng AL, Kokudo N, Kudo M, Lee JM, Jia J, et al. Asia-Pacific clinical practice guidelines on the management of hepatocellular carcinoma: a 2017 update. Hepatol Int. 2017;11(4):317-70.

3. European Association for the Study of the Liver. Electronic address eee, European Association for the Study of the L. EASL Clinical Practice Guidelines: Management of hepatocellular carcinoma. J Hepatol. 2018;69(1):182-236.

4. Heimbach JK, Kulik LM, Finn RS, Sirlin CB, Abecassis MM, Roberts LR, et al. AASLD guidelines for the treatment of hepatocellular carcinoma. Hepatology. 2018;67(1):358-80.

5. Shin SW, Ahn KS, Kim SW, Kim TS, Kim YH, Kang KJ. Liver Resection Versus Local Ablation Therapies for Hepatocellular Carcinoma Within the Milan Criteria: A Systematic Review and Meta-analysis. Ann Surg. 2021;273(4):656-66.

6. Xu X-L, Liu X-D, Liang M, Luo B-M. Radiofrequency Ablation versus Hepatic Resection for Small Hepatocellular Carcinoma: Systematic Review of Randomized Controlled Trials with Meta-Analysis and Trial Sequential Analysis. Radiology. 2017:162756.

7. Lee MW, Kang D, Lim HK, Cho J, Sinn DH, Kang TW, et al. Updated 10-year outcomes of percutaneous radiofrequency ablation as first-line therapy for single hepatocellular carcinoma < 3 cm: emphasis on association of local tumor progression and overall survival. Eur Radiol. 2020;30(4):2391-400.

8. Kim YS, Lim HK, Rhim H, Lee MW, Choi D, Lee WJ, et al. Ten-year outcomes of percutaneous radiofrequency ablation as first-line therapy of early hepatocellular carcinoma: analysis of prognostic factors. J Hepatol. 2013;58(1):89-97.

9. Kim YS, Lee WJ, Rhim H, Lim HK, Choi D, Lee JY. The minimal ablative margin of radiofrequency ablation of hepatocellular carcinoma (> 2 and < 5 cm) needed to prevent local tumor progression: 3D quantitative assessment using CT image fusion. AJR Am J Roentgenol. 2010;195(3):758-65.

10. Yoon JH, Lee JM, Klotz E, Woo H, Yu MH, Joo I, et al. Prediction of Local Tumor Progression after Radiofrequency Ablation (RFA) of Hepatocellular Carcinoma by Assessment of Ablative Margin Using Pre-RFA MRI and Post-RFA CT Registration. Korean J Radiol. 2018;19(6):1053-65.

11. Ahmed M, Solbiati L, Brace CL, Breen DJ, Callstrom MR, Charboneau JW, et al. Image-guided tumor ablation: standardization of terminology and reporting criteria--a 10-year update. Radiology. 2014;273(1):241-60.

12. Sasaki A, Kai S, Iwashita Y, Hirano S, Ohta M, Kitano S. Microsatellite distribution and indication for locoregional therapy in small hepatocellular carcinoma. Cancer. 2005;103(2):299-306.

13. Ausania F, Borin A, Melendez R, Rio PSD, Iglesias A, Bodenlle P, et al. Microwave ablation of colorectal liver metastases: Impact of a 10-mm safety margin on local recurrence in a tertiary care hospital. Ann Hepatobiliary Pancreat Surg. 2021;25(3):366-70.

14. Lachenmayer A, Tinguely P, Maurer MH, Frehner L, Knopfli M, Peterhans M, et al. Stereotactic image-guided microwave ablation of hepatocellular carcinoma using a computer-assisted navigation system. Liver Int. 2019;39(10):1975-85.

15. Sakon M, Nagano H, Nakamori S, Dono K, Umeshita K, Murakami T, et al. Intrahepatic recurrences of hepatocellular carcinoma after hepatectomy: analysis based on tumor hemodynamics. Arch Surg. 2002;137(1):94-9.

16. Park JH, Koh KC, Choi MS, Lee JH, Yoo BC, Paik SW, et al. Analysis of risk factors associated with early multinodular recurrences after hepatic resection for hepatocellular carcinoma. Am J Surg. 2006;192(1):29-33.

17. Nakashima Y, Nakashima O, Tanaka M, Okuda K, Nakashima M, Kojiro M. Portal vein invasion and intrahepatic micrometastasis in small hepatocellular carcinoma by gross type. Hepatol Res. 2003;26(2):142-7.

18. Kudo M, Kawamura Y, Hasegawa K, Tateishi R, Kariyama K, Shiina S, et al. Management of Hepatocellular Carcinoma in Japan: JSH Consensus Statements and Recommendations 2021 Update. Liver Cancer. 2021;10(3):181-223.

19. Choi JY, Lee JM, Sirlin CB. CT and MR imaging diagnosis and staging of hepatocellular carcinoma: part I. Development, growth, and spread: key pathologic and imaging aspects. Radiology. 2014;272(3):635-54.

20. Ueda K, Matsui O, Kawamori Y, Nakanuma Y, Kadoya M, Yoshikawa J, et al. Hypervascular hepatocellular carcinoma: evaluation of hemodynamics with dynamic CT during hepatic arteriography. Radiology. 1998;206(1):161-6.

21. Imura S, Yamada S, Saito Y, Ikemoto T, Morine Y, Shimada M. Utility of cone unit liver resection for small hepatocellular carcinoma: a propensity score matched analysis. HPB (Oxford). 2021;23(5):739-45.

22. Kang KJ, Ahn KS. Anatomical resection of hepatocellular carcinoma: A critical review of the procedure and its benefits on survival. World J Gastroenterol. 2017;23(7):1139-46.

23. Haruki K, Furukawa K, Fujiwara Y, Taniai T, Hamura R, Shirai Y, et al. Effectiveness of Anatomical Resection for Small Hepatocellular Carcinoma: a Propensity Score-Matched Analysis of a Multi-institutional Database. J Gastrointest Surg. 2021;25(11):2835-41.

24. Ueno S, Kubo F, Sakoda M, Hiwatashi K, Tateno T, Mataki Y, et al. Efficacy of anatomic resection vs nonanatomic resection for small nodular hepatocellular carcinoma based on gross classification. J Hepatobiliary Pancreat Surg. 2008;15(5):493-500.

25. Luerken L, Haimerl M, Doppler M, Uller W, Beyer LP, Stroszczynski C, et al. Update on Percutaneous Local Ablative Procedures for the Treatment of Hepatocellular Carcinoma. Rofo. 2022.

26. Lee DH, Lee JM. Recent Advances in the Image-Guided Tumor Ablation of Liver Malignancies: Radiofrequency Ablation with Multiple Electrodes, Real-Time Multimodality Fusion Imaging, and New Energy Sources. Korean J Radiol. 2018;19(4):545-59.

27. Tamai H, Okamura J. New next-generation microwave thermosphere ablation for small hepatocellular carcinoma. Clin Mol Hepatol. 2021;27(4):564-74.

28. Li K, Long Y, He X, Wu Y, Xu J, Ye H, et al. Comparison of anatomical thermal ablation and routine thermal ablation for hepatocellular carcinoma</=50 mm: A propensity score matching. Hepatol Res. 2022;52(7):641-51.

29. Korean Liver Cancer A, National Cancer Center GK. 2018 Korean Liver Cancer Association-National Cancer Center Korea Practice Guidelines for the Management of Hepatocellular Carcinoma. Korean J Radiol. 2019;20(7):1042-113.

30. Lee DH, Lee MW, Kim PN, Lee YJ, Park HS, Lee JM. Outcome of No-Touch Radiofrequency Ablation for Small Hepatocellular Carcinoma: A Multicenter Clinical Trial. Radiology. 2021;301(1):229-36.

31. Kang HJ, Lee JM, Jeon SK, Ryu H, Yoo J, Lee JK, et al. Microvascular Flow Imaging of Residual or Recurrent Hepatocellular Carcinoma after Transarterial Chemoembolization: Comparison with Color/Power Doppler Imaging. Korean J Radiol. 2019;20(7):1114-23.

32. Park SJ, Cho EJ, Lee JH, Yu SJ, Kim YJ, Yoon JH, et al. Switching Monopolar No-Touch Radiofrequency Ablation Using Octopus Electrodes for Small Hepatocellular Carcinoma: A Randomized Clinical Trial. Liver Cancer. 2021;10(1):72-81.

33. Suh YS, Choi JW, Yoon JH, Lee DH, Kim YJ, Lee JH, et al. No-Touch vs. Conventional Radiofrequency Ablation Using Twin Internally Cooled Wet Electrodes for Small Hepatocellular Carcinomas: A Randomized Prospective Comparative Study. Korean J Radiol. 2021;22(12):1974-84.

34. Choi JW, Lee JM, Lee DH, Yoon JH, Kim YJ, Lee JH, et al. Radiofrequency Ablation Using a Separable Clustered Electrode for the Treatment of Hepatocellular Carcinomas: A Randomized Controlled Trial of a Dual-Switching Monopolar Mode Versus a Single-Switching Monopolar Mode. Korean J Radiol. 2021;22(2):179-88.

35. Mauri G, Cova L, De Beni S, Ierace T, Tondolo T, Cerri A, et al. Real-time US-CT/MRI image fusion for guidance of thermal ablation of liver tumors undetectable with US: results in 295 cases. Cardiovasc Intervent Radiol. 2015;38(1):143-51.

36. Clavien PA, Barkun J, de Oliveira ML, Vauthey JN, Dindo D, Schulick RD, et al. The Clavien-Dindo classification of surgical complications: five-year experience. Ann Surg. 2009;250(2):187-96.

37. Puijk RS, Ahmed M, Adam A, Arai Y, Arellano R, de Baere T, et al. Consensus Guidelines for the Definition of Time-to-Event End Points in Image-guided Tumor Ablation: Results of the SIO and DATECAN Initiative. Radiology. 2021;301(3):533-40.

38. Morimoto M, Numata K, Sugimori K, Shirato K, Kokawa A, Oka H, et al. Successful initial ablation therapy contributes to survival in patients with hepatocellular carcinoma. World J Gastroenterol. 2007;13(7):1003-9.

39. Iwamoto S, Yamaguchi T, Hongo O, Iwamoto H, Sanefuji H. Excellent outcomes with angiographic subsegmentectomy in the treatment of typical hepatocellular carcinoma: a retrospective study of local recurrence and long-term survival rates in 120 patients with hepatocellular carcinoma. Cancer. 2010;116(2):393-9.

40. Miyayama S, Yamashiro M, Ikuno M, Okumura K, Yoshida M. Ultraselective transcatheter arterial chemoembolization for small hepatocellular carcinoma guided by automated tumor-feeders detection software: technical success and short-term tumor response. Abdom Imaging. 2014;39(3):645-56.

41. Spiliotis AE, Gabelein G, Hollander S, Scherber PR, Glanemann M, Patel B. Microwave ablation compared with radiofrequency ablation for the treatment of liver cancer: a systematic review and meta-analysis. Radiol Oncol. 2021;55(3):247-58.

42. Lee MW. Fusion imaging of real-time ultrasonography with CT or MRI for hepatic intervention. Ultrasonography. 2014;33(4):227-39.

43. Ahn SJ, Lee JM, Lee DH, Lee SM, Yoon JH, Kim YJ, et al. Real-time US-CT/MR fusion imaging for percutaneous radiofrequency ablation of hepatocellular carcinoma. J Hepatol. 2017;66(2):347-54.

44. Song KD, Kim SH, Lim HK, Jung SH, Sohn I, Kim HS. Subcentimeter hypervascular nodule with typical imaging findings of hepatocellular carcinoma in patients with history of hepatocellular carcinoma: natural course on serial gadoxetic acid-enhanced MRI and diffusion-weighted imaging. Eur Radiol. 2015;25(9):2789-96.

45. Wang X, Sofocleous CT, Erinjeri JP, Petre EN, Gonen M, Do KG, et al. Margin size is an independent predictor of local tumor progression after ablation of colon cancer liver metastases. Cardiovasc Intervent Radiol. 2013;36(1):166-75.

46. Joo I, Morrow KW, Raman SS, McWilliams JP, Sayre JW, Lu DS. Correction to: CT-monitored minimal ablative margin control in single-session microwave ablation of liver tumors: an effective strategy for local tumor control. Eur Radiol. 2022.

47. Petit A, Hocquelet A, N'Kontchou G, Varin E, Sellier N, Seror O, et al. No-Touch Multi-bipolar Radiofrequency Ablation for the Treatment of Subcapsular Hepatocellular Carcinoma </= 5 cm Not Puncturable via the Non-tumorous Liver Parenchyma. Cardiovasc Intervent Radiol. 2019.

48. Qu C, Li XQ, Li C, Xia F, Feng K, Ma K. The Short-Term Efficacy of Novel No-Touch Combined Directional Perfusion Radiofrequency Ablation in the Treatment of Small Hepatocellular Carcinoma with Cirrhosis. Journal of investigative surgery : the official journal of the Academy of Surgical Research. 2021:1-8.

49. Seror O, N'Kontchou G, Nault JC, Rabahi Y, Nahon P, Ganne-Carrie N, et al. Hepatocellular Carcinoma within Milan Criteria: No-Touch Multibipolar Radiofrequency Ablation for Treatment-Long-term Results. Radiology. 2016;280(2):611-21.

50. Wu L-W, Chen C-Y, Liu C-J, Chen M-Y, Liu P-C, Liu P-F, et al. Multipolar radiofrequency ablation with non-touch technique for hepatocellular carcinoma ≤ 3 cm: A preliminary report. Advances in Digestive Medicine. 2014;1(3):80-5.

51. Kang TW, Lim HK, Lee MW, Kim YS, Rhim H, Lee WJ, et al. Aggressive Intrasegmental Recurrence of Hepatocellular Carcinoma after Radiofrequency Ablation: Risk Factors and Clinical Significance. Radiology. 2015;276(1):274-85.

52. Tung-Ping Poon R, Fan ST, Wong J. Risk factors, prevention, and management of postoperative recurrence after resection of hepatocellular carcinoma. Ann Surg. 2000;232(1):10-24.

**TABLES**

Table 1. Participants Characteristics

| Characteristic | Data |
| --- | --- |
| Age (y) | 66.4±7.7 |
| Sex |  |
| No. of men | 39 (70.9) |
| No. of women | 16 (29.1) |
| Underlying liver disease |  |
| Hepatitis B virus | 43 (78.2) |
| Hepatitis C virus | 6 (10.9) |
| Alcohol | 6 (10.9) |
| Child-Pugh class |  |
| A | 55 (100) |
| B | 0 (0) |
| Tumor size (cm) | 1.77±0.59 |
| AFP (ng/mL) | 58.2±239.0 |
| Tumor location |  |
| Right anterior section | 18 (32.7) |
| Right posterior section | 25 (45.5) |
| Left medial section | 2 (3.6) |
| Left lateral section | 10 (18.2) |
| Ablation zone size (cm) | 5.34±0.95 |

Note.—Data are mean ± standard deviation or n (%).

**FIGURE LEGENDS**

Figure 1. Schematic illustration of anatomical ablation. (a) One applicator is placed across the tumor-bearing 5^th^ portal vein branch. The other applicators are placed peritumoral zone. (b) The ablation zone covers all the tumor-bearing portal vein territory with a sufficient safety margin. (c) Small satellite nodules or microvascular invasion are located along the portal vein branches that drain from the primary tumor. (d) Anatomical ablation might effectively remove both the primary tumor and those satellite nodules or microvascular invasion.

Figure 2. Flow diagram of included patients.

Figure 3. Anatomical radiofrequency ablation in a 70-year-old woman with a 2.0-cm hepatocellular carcinoma (HCC) and hepatitis B-related cirrhosis. (A) Hepatobiliary phase image of gadoxetic acid-enhanced MRI shows a 2.0-cm low signal intensity HCC in segment VI of the liver. Two tumor-bearing portal vein 4th branches are noted (arrows). (B) Contrast-enhanced ultrasound (Sonazoid) images well visualize the tumor (large arrows) and tumor-bearing portal vein branches (small arrows). (C) Real-time US-MR fusion image shows the low echoic target tumor (large arrow) and electrode placed across the tumor-bearing portal vein branches (small arrows). (D) Portal venous phase coronal image of immediate CT scan shows complete ablation of the target tumor and tumor-bearing portal vein branches with a sufficient safety margin (>5 mm). (E) No local tumor progression was observed at 21-month follow-up CT.

Figure 4. Technical failure of anatomical radiofrequency ablation in a 60-year-old man with a 2.0-cm hepatocellular carcinoma (HCC) and hepatitis B-related cirrhosis. (A) Hepatobiliary phase image of gadoxetic acid enhanced MRI shows a 2.0-cm low signal intensity HCC in segment VIII of the liver (large arrow). The 4^th^ order portal vein branch close to the medial border of the tumor is visualized (small arrows). (B) Portal venous phase coronal reformatted image of immediate CT scan shows an insufficient medial safety margin (<5 mm). Ablation of the 4^th^ order portal vein branch (arrows) located near the medial border of the tumor had failed. (C) Arterial phase coronal reformatted image of 6-month follow-up CT shows a recurrent tumor with arterial enhancement at the medial aspect of the ablation defect (arrow). (D) Portal venous phase coronal reformatted image of 6-month follow-up CT shows a connection between the recurrent tumor (large arrow) and remnant 4^th^ order portal vein branch (small arrows), which was not ablated.

Figure 5. Kaplan–Meier estimation of the cumulative incidence of (a) local tumor progression and (b) recurrence-free survival after anatomical radiofrequency ablation in a single hepatocellular carcinoma ≤4.0 cm.
